# Influence of adjuvant chemotherapy on survival for patients with stage IB and IIA non‐small cell lung cancer

**DOI:** 10.1111/1759-7714.13685

**Published:** 2020-10-27

**Authors:** Pei Zhang, Jianchun Duan, Hua Bai, Zhijie Wang, Shugeng Gao, Fengwei Tan, Yushun Gao, Xin Wang, Rui Wan, Jiachen Xu, Xiran He, Xiaoshuang Feng, Ruofei Yu, Jing Sun, Zhe Zhao, Kailun Fei, Ni Li, Jie He, Jie Wang

**Affiliations:** ^1^ Department of Medical Oncology, National Cancer Center/National Clinical Research Center for Cancer/Cancer Hospital Chinese Academy of Medical Sciences & Peking Union Medical College Beijing China; ^2^ Department of Thoracic Surgery, National Cancer Center/National Clinical Research Center for Cancer/Cancer Hospital Chinese Academy of Medical Sciences & Peking Union Medical College Beijing China; ^3^ Office of Cancer Screening, National Cancer Center/National Clinical Research Center for Cancer/Cancer Hospital Chinese Academy of Medical Sciences and Peking Union Medical College Beijing China

**Keywords:** Adjuvant chemotherapy, eighth edition of the AJCC TNM staging system, stage IB–IIA NSCLC

## Abstract

**Background:**

The role of adjuvant chemotherapy (ACT) for patients with stage IB–IIA non‐small cell lung cancer (NSCLC) according to the eighth edition of the AJCC TNM staging system remains controversial.

**Methods:**

Data were collected from patients with NSCLC stage IB–IIA according to the eighth edition of the AJCC TNM staging system who underwent surgical resection from 2008 to 2015. The relationship between ACT and overall survival (OS) or disease‐free survival (DFS) was analyzed using the Kaplan–Meier method and Cox proportional hazards model.

**Results:**

The study included 648 patients with completely resected NSCLC stage IB–IIA; 312 underwent ACT after surgical resection and 336 were placed under observation. After propensity score matching, 247 pairs of patients were matched and the five‐year OS was 88.08% and 83.12% (*P* = 0.13) in ACT and non‐ACT settings, respectively. Subgroup analyses demonstrated that ACT treatment was correlated with an improved five‐year OS in patients with visceral pleural invasion (VPI) in the 3 < tumor ≤ 4 cm subgroup (93.98% and 68.93%, *P* < 0.01).

**Conclusions:**

ACT was not significantly associated with improved five‐year OS in stage IB–IIA NSCLC patients. However, further subgroup analysis showed that patients with VPI in the 3 < tumor ≤ 4 cm (T2aN0M0, stage IB) subgroup might benefit more from ACT. Further studies are required to validate the findings and better systemic strategies need to be developed in these patients.

**Key points:**

**Significant findings of the study:**

- For patients with stage IB–IIA NSCLC according to the eighth edition of the AJCC TNM staging system, the effect of ACT remains unclear.
- ACT was not significantly associated with improved five‐year OS in stage IB–IIA NSCLC patients. However, it was correlated with better DFS before or after PSM.
- Patients with VPI in the 3 < tumor ≤ 4 cm subgroup may benefit from ACT.

**What this study adds:**

- ACT was not significantly associated with improved five‐year OS in stage IB–IIA NSCLC patients. However, it was correlated with better DFS before or after PSM.
- Patients with VPI in the 3 < tumor ≤ 4 cm subgroup may benefit from ACT.

## Graphical abstract

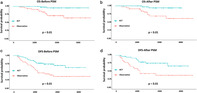

Whether adjuvant chemotherapy can improve the survival of patients with stage IB–IIA NSCLC, according to the eighth edition of the AJCC TNM staging system, remains unclear. We reviewed our database and found that patients with VPI in the 3 < tumor ≤ 4 cm subgroup may benefit from ACT.

## Introduction

Lung cancer is one of the most commonly diagnosed cancers (11.6% of total cases) and the leading cause of cancer death (18.4% of total cancer deaths) predicted in 2018.^1^ Non‐small cell lung cancer (NSCLC) is the primary histological type accounting for approximately 85% of lung cancers.^2^ Despite complete resection, a large proportion of patients remain at risk of tumor recurrence. Even in early stage NSCLC, certain patients experience local recurrence and distant metastasis due to micrometastasis before surgical resection.^3^, ^4^, ^5^

Randomized controlled trials and meta‐analyses of adjuvant chemotherapy (ACT) versus observation in patients with resected NSCLC have previously demonstrated a statistically significant benefit of the addition of systemic therapy.^6^, ^7^, ^8^, ^9^, ^10^ Moreover, the LACE pooled analysis included a total of 4584 patients accrued in five adjuvant trials (ALPI‐EORTC, IALT, JBR.10, ANITA, and Big Lung Trial). It confirmed that there was an improvement in five‐year overall survival (OS) and disease‐free survival (DFS) when ACT was administered in patients with stage II–IIIA NSCLC who underwent complete resection, especially in those where cisplatin plus vinorelbine was used as ACT.^4^, ^6^, ^7^, ^8^, ^11^, ^12^ Therefore, ACT has been a standard treatment for stage II–IIIA NSCLC patients in the setting of complete resection.

However, the efficacy of ACT on patients with stage IB NSCLC remains controversial. In the Cancer and Leukemia Group B (CALBG) 9633 trial, patients with stage IB NSCLC (tumor > 3 cm; sixth edition of the TNM classification) failed to show an overall survival (OS) benefit with ACT, except in patients with tumor > 4 cm.^9^, ^13^ Interestingly, a large retrospective analysis of the National Cancer Data Base showed that ACT was associated with improved OS in stage IB NSCLC patients (tumor 3–7 cm; seventh edition of the TNM classification). In this study, even those patients with smaller tumors (tumor 3–4 cm) could benefit from ACT.^14^ In 2017, the eighth edition of the TNM classification was adopted worldwide. Compared to the seventh edition, some criteria for T classifications were changed.^15^ T2a was previously considered to be a tumor with a size ranging from 3 to 5 cm. However, this range was divided into two parts in the eighth edition: one ranging from 3 to 4 cm (T2aN0M0, stage IB) and the other from 4 to 5 cm (T2bN0M0, stage IIA). Because of these changes, the early stage NSCLC population that should be considered for chemotherapy after surgery requires refinement.

In the current study, we investigated 648 cases of stage IB–IIA NSCLC who underwent surgical resection in the Cancer Hospital Chinese Academy of Medical Sciences from 2008 to 2015. Relative clinical and pathological characteristics that may influence survival results were collected, including sex, age, smoking history, histopathological diagnosis, tumor size, differentiation, lympovascular invasion (LVI), visceral pleural involvement (VPI), and number of examined lymph nodes (ELNs). The aim of this study was to investigate whether ACT can improve the OS for patients with stage IB–IIA NSCLC according to the eighth edition of the TNM classification.

## Methods

### Patients and adjuvant chemotherapy

A total of 648 pathological stage IB–IIA NSCLC patients who underwent surgical resection at the National Cancer Center/National Clinical Research Center for Cancer/Cancer Hospital Chinese Academy of Medical Sciences/Peking Union Medical College between 1 January 2008, and 30 April 2015, were included in this study. Pathological staging was done according to the eighth edition of the TNM classification. In the eighth edition of the TNM classification, T2 is considered to be a tumor >3 cm but ≤5 cm, or having any of the following features: (i) Involving the main bronchus, regardless of distance to the carina, but without involvement of the carina; (ii) invading the visceral pleura (PL1 or PL2); and (iii) is associated with atelectasis or obstructive pneumonitis that extends to the hilar region, involving part or all of the lung. Therefore, we included the cases with these features in the 0 < tumor ≤ 3 cm group (T2N0M0, stage IB). Moreover, T2aN0M0 was defined as 3 < tumor ≤ 4 cm (stage IB) and T2bN0M0 was 4 < tumor ≤ 5 cm (stage IIA). We excluded data from patients with neoadjuvant chemotherapy, radiotherapy, wedge excision, positive surgical margins, recurrence within six months, or death within one month.

Patients were included if they had received ACT within 1–3 months after surgical resection. The decision as to whether a patient received or did not receive ACT was based on the physical and pathological condition of each patient. ACT consisted mostly of platinum‐based agents. Most regimens included cisplatin or carboplatin combined with vinorelbine, gemcitabine, pemetrexed, paclitaxel, or docetaxel. Due to physical conditions or personal wishes, some patients received only single‐agent chemotherapy without platinum agents. Four cycles of ACT were routinely administered.

### Follow‐up

Routine surveillance after completion of definitive therapy, including history and physical examination, blood tests, and chest CT scan, were done every 3–6 months for the first three years, at six‐month intervals for the next two years, and annually thereafter. Once symptoms or signs of recurrence appeared, patients underwent systemic examinations. The duration of OS was defined as the interval between the date of surgical resection and death. The duration of DFS was defined as the interval between the date of surgical resection and locoregional or distant recurrence.

### Statistical analysis

The Student's *t*‐test and Pearson's Chi‐square test were used to compare clinical and pathological characteristics between ACT and observation groups. To reduce selection bias, propensity score matching (PSM) was used to achieve balance in baseline characteristics between the ACT and observation groups. Patients were matched regard to sex, age, smoking history, histopathological diagnosis, tumor size, tumor differentiation, LVI, VPI, and ELNs.

The Kaplan–Meier method with log‐rank test was used to estimate OS and DFS rates in all patients and in such subgroups. Univariate and multivariate regression models were constructed to estimate the simultaneous effects of potential independent predictors of survival. Multivariate regression models incorporated factors identified in univariate analyses with *P*‐values <0.05.

All data were double‐entered and then exported to tab‐delimited text files. All analyses were performed with R (http://www.R-project.org) and EmpowerStats software (www.empowerstats.com, X&Y solutions, Inc., Boston, MA).

## Results

### **Clinicopathological characteristics of patients with stage IB–IIA** **NSCLC**

This study included 648 patients, comprising 566 with pathological stage IB disease and 82 with stage IIA (4 < tumor ≤5 cm) disease according to the eighth edition of the staging system. Patients with stage IB disease were divided into two subgroups based on tumor size (403 cases in the 0 < tumor ≤ 3 cm group, 163 cases in the 3 < tumor ≤ 4 cm group). Among these patients, 312 (48.15%) underwent ACT after surgical resection and 336 (51.85%) were placed under observation. ACT was more common in the subgroup patients with young age, smoking history, larger tumor size, poor differentiation or LVI (*P* < 0.05). After PSM, 247 pairs of patients were individually matched between the two groups, and all baseline characteristics were well‐balanced. Table 1 summarizes the demographic and clinical characteristics of patients, including etiology, surgical procedure and risk factors.

**Table 1:** Clinicopathological characteristics of patients before and after propensity score matching (PSM)

	Before PSM			After PSM		
	Observation	ACT	*P*‐value	Observation	ACT	*P*‐value
N	336	312		247	247	
Age	(336) 61.32 ± 9.17	(312) 57.16 ± 8.76	**<0.01**	(247) 59.65 ± 8.98	(247) 58.15 ± 8.71	0.19
Sex			0.34			0.53
Female	159 (47.2%)	136 (43.59%)		118 (47.77%)	111 (44.94%)	
Male	177 (52.68%)	176 (47.76%)		129 (52.23%)	136 (55.06%)	
Smoking history			**0.02**			0.27
No	205 (61.01%)	163 (52.24%)		150 (60.73%)	138 (55.87%)	
Yes	131 (38.99%)	149 (47.76%)		97 (39.27%)	109 (44.13%)	
Histology			0.63			0.09
Nonadenocarcinoma	66 (19.64%)	66 (21.15%)		40 (16.19%)	55 (22.27%)	
Adenocarcinoma	270 (80.36%)	246 (78.85%)		207 (83.81%)	192 (77.73%)	
Differentiation			**<0.01**			0.16
High and moderate	275 (81.85%)	194 (62.18%)		188 (76.11%)	174 (70.45%)	
Poor	61 (18.15%)	118 (37.81%)		59 (23.89%)	73 (29.55%)	
Tumor size			**<0.01**			0.17
0 < tumor ≤ 3 cm	224 (66.67%)	179 (57.73%)		168 (68.02%)	151 (61.13%)	
3 < tumor ≤ 4 cm	83 (24.70%)	80 (25.64%)		56 (22.67%)	61 (24.70%)	
4 < tumor ≤ 5 cm	29 (8.63%)	53 (16.99%)		23 (9.31%)	35 (14.17%)	
VPI			0.48			0.19
No	53 (15.77%)	43 (13.78%)		29 (11.74%)	39 (15.79%)	
Yes	283 (84.23%)	269 (86.22%)		218 (88.26%)	208 (84.21%)	
ELNs			0.31			0.68
≥10	287 (85.42%)	275 (88.14%)		215 (87.04%)	218 (88.26%)	
<10	49 (14.58%)	37 (11.86%)		32 (12.96%)	29 (11.74%)	
LVI			**<0.01**			0.39
No	319 (94.94%)	279 (89.42%)		231 (93.52%)	226 (91.50%)	
Yes	17 (5.06%)	33 (10.58%)		16 (6.48%)	21 (8.50%)	

Bold numbers indicate statistical significance (*P* < 0.05). ACT, adjuvant chemotherapy; ELNs, examined lymph nodes; LVI, lymphovascular invasion; N, number; PSM, propensity score matching: VPI, visceral pleural involvement.

After univariate regression analyses and multivariate risk adjustment for potential confounding factors, we found that larger tumor size (4 < tumor ≤ 5 cm) (hazard ratio [HR], 2.48; 95% confidence interval [CI]: 1.54–4.02; *P* < 0.01) was an independent adverse predictor of OS **(**Table 2 **)**. Post‐PSM data further corroborated this conclusion (HR, 2.10; 95% CI: 1.14–3.87; *P* = 0.02). Irrespective of whether this was before or after PSM analyses, large tumor size was found to be associated with poor DFS (HR, 1.72; 95% CI: 1.22–2.42; *P* < 0.01; HR, 1.99; 95% CI: 1.35–2.93; *P* < 0.01) **(**Table 2 **)**. Despite the insufficient number of cases, patients with larger tumor sizes (4 < tumor ≤ 5 cm) had significantly worse OS than those with smaller tumors (0 < tumor ≤3 cm; 3 < tumor ≤ 4 cm). The five‐year OS and DFS rates of patients with larger tumors were 71.02% and 44.68%, respectively, which was significantly lower than those of patients with small tumors (0 < tumor ≤ 3 cm group: 86.04% and 61.92%; 3 < tumor ≤ 4 cm group: 87.32% and 62.55%). Subgroup analysis stratified by tumor size was also performed in the matched population (SI1). Furthermore, having fewer ELNs (<10) was associated with a higher recurrence rate (HR, 1.51; 95% CI: 1.10–2.09; *P* = 0.01). Unfortunately, we did not detect a relationship between fewer ELNs and shorter DFS after PSM **(**Table 2 **)**.

**Table 2:** Univariable and multivariable analyses of overall survival (OS) and disease‐free survival (DFS) before or after propensity score matching (PSM)

	Before PSM						After PSM					
	Univariate			Multivariate			Univariate			Multivariate		
	HR	95% CI	*P*‐value	HR	95% CI	*P*‐value	HR	95% CI	*P*‐value	HR	95% CI	*P*‐value
OS												
ACT	0.68	0.45–1.02	0.06				0.70	0.43–1.12	0.13			
Age	1.05	1.03–1.08	**<0.01**	1.05	1.03–1.08	**<0.01**	1.04	1.01–1.07	**<0.01**	1.04	1.01–1.07	**<0.01**
Male	1.32	0.88–1.98	0.17				1.47	0.91–2.36	0.12			
Smoking history	1.10	0.74–1.64	0.64				1.41	0.88–2.25	0.15			
Adenocarcinoma	1.08	0.65–1.78	0.77				1.32	0.69–2.51	0.40			
Poor differentiation	0.75	0.46–1.23	0.26				0.87	0.50–1.52	0.63			
3 < tumor ≤ 4 cm	0.89	0.53–1.50	0.66				1.12	0.63–2.00	0.69			
4 < tumor ≤ 5 cm	2.73	1.69–4.42	**<0.01**	2.48	1.54–4.02	**<0.01**	2.33	1.27–4.28	**<0.01**	2.10	1.14–3.87	**0.02**
VPI	1.03	0.59–1.82	0.92				0.94	0.48–1.84	0.86			
ELNs < 10	1.65	1.00–2.73	0.05				1.62	0.89–2.95	0.12			
LVI	0.95	0.41–2.16	0.89				0.62	0.19–1.96	0.41			
DFS												
ACT	0.76	0.60–0.97	0.03	0.78	0.61–1.00	0.05	0.76	0.58–1.00	**<0.05**	0.75	0.57–0.98	**0.04**
Age	1.02	1.01–1.04	**<0.01**	1.02	1.01–1.03	**0.01**	1.02	1.00–1.03	**0.04**	1.01	1.00–1.03	0.12
Male	1.19	0.93–1.51	0.16				1.18	0.90–1.55	0.23			
Smoking history	1.05	0.83–1.34	0.67				1.16	0.88–1.53	0.29			
Adenocarcinoma	1.15	0.84–1.56	0.38				1.20	0.84–1.72	0.32			
Poor differentiation	1.15	0.88–1.50	0.31				1.30	0.96–1.74	0.09			
3 < tumor ≤ 4 cm	1.08	0.81–1.43	0.62				1.03	0.74–1.45	0.85			
4 < tumor ≤ 5 cm	1.76	1.25–2.49	**<0.01**	1.72	1.22–2.42	**<0.01**	2.00	1.36–2.93	**<0.01**	1.99	1.35–2.93	**<0.01**
VPI	0.98	0.70–1.38	0.92				0.99	0.67–1.47	0.96			
ELN < 10	1.49	1.08–2.05	**0.02**	1.51	1.10–2.09	**0.01**	1.30	0.89–1.90	0.18			
LVI	1.31	0.85–2.01	0.22				1.31	0.81–2.12	0.27			

Bold numbers indicate statistical significance (*P*< 0.05). ACT, adjuvant chemotherapy; CI, confidence interval; ELNs, examined lymph nodes; HR, hazard ratio; LVI, lymphovascular invasion; N, number; PSM, propensity score matching: VPI, visceral pleural involvement.

### Comparison of five‐year survival rates between patients receiving ACT and those placed under observation

**Figure 1:**
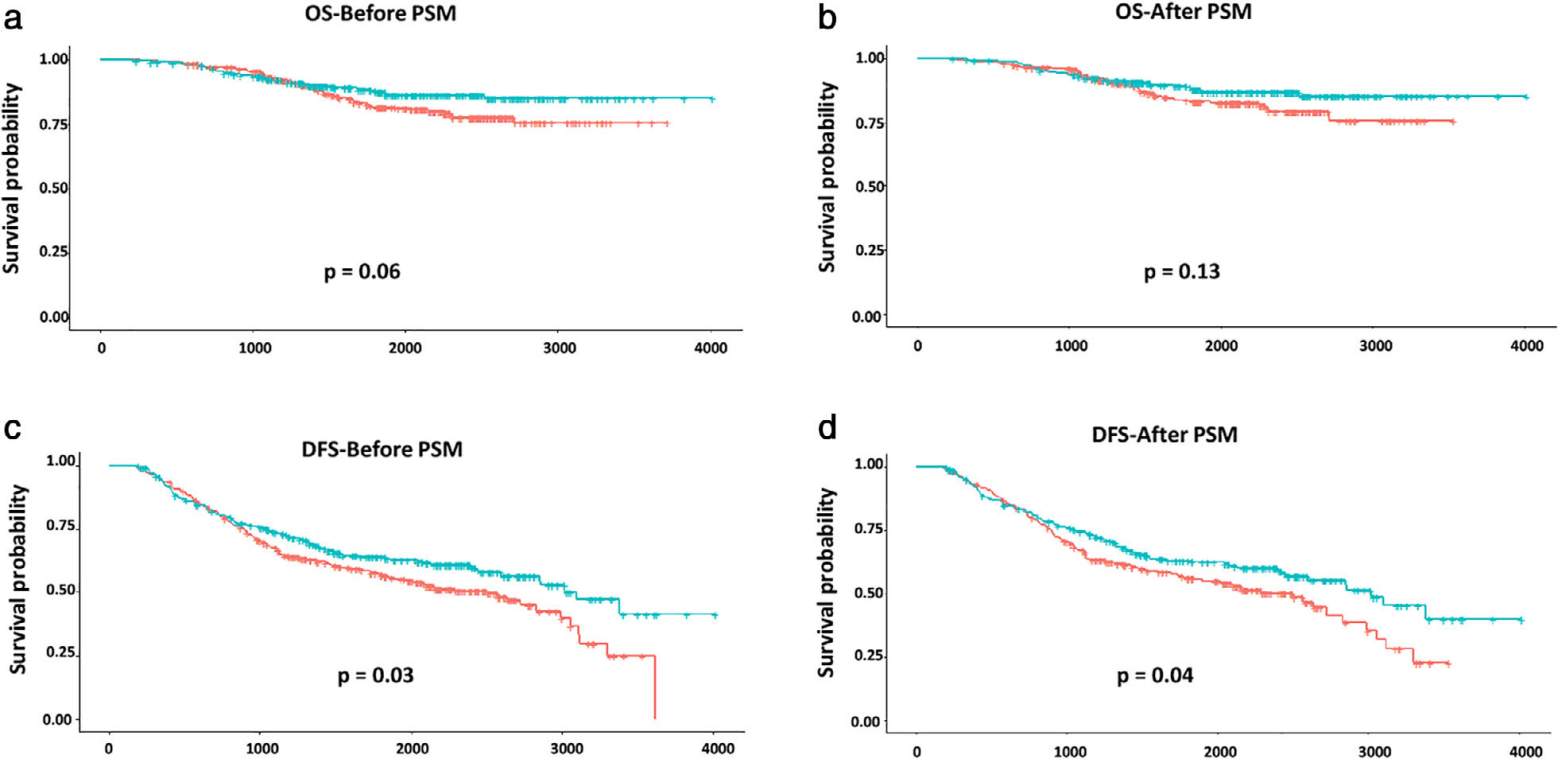
Kaplan‐Meier survival curves of OS and DFS for patients who received adjuvant chemotherapy (ACT) before or after propensity score matching (PSM). (**a**,**b**) Kaplan‐Meier survival curves of OS for patients who received ACT before or after PSM 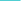, ACT; 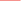, observation; 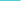, ACT; 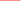, observation. (**c**,**d**) Kaplan‐Meier survival curves of DFS for patients who received ACT before or after PSM 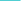, ACT; 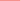, observation; 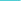, ACT; 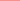, observation. ACT, adjuvant chemotherapy; DFS, disease‐free survival; OS, overall survival; PSM, propensity score matching.

With a median follow‐up of 63.72 months (range, 6.90–133.77 months), the five‐year OS rate was 84.54% for all study participants as determined using Kaplan–Meier survival models and log‐rank tests. However, subgroup analysis showed that ACT was not associated with higher survival rate in patients with stage IB–IIA NSCLC. The five‐year OS rates were 87.66% and 81.65% for patients in the ACT and observation groups, respectively (*P* = 0.06) **(**Fig 1a **)**. After PSM was performed, the five‐year survival rates in patients with ACT and those placed under observation were statistically similar at 88.08% and 83.12%, respectively (*P* = 0.13) **(**Fig 1b **)**. Although ACT did not demonstrate a benefit in OS, it was correlated with better DFS before or after PSM (63.90% vs. 56.33%, *P* = 0.03; 65.14% vs. 56.60%, *P* = 0.04) (Fig 1c,d).

Furthermore, subgroup analyses showed that patients with VPI may gain a DFS benefit from ACT both before and after PSM (HR, 0.69; 95% CI: 0.51–0.93; *P* = 0.01; HR, 0.70; 95% CI: 0.53–0.95; *P* = 0.02, respectively). However, there was no ACT influence on OS in such patients (Table 3).

**Table 3:** Association between adjuvant chemotherapy (ACT) and survival of stage IB–IIA patients according to baseline characteristics before or after propensity score matching (PSM)

	Before PSM				After PSM			
	N	HR	95% CI	*P*‐value	N	HR	95% CI	*P*‐value
OS								
Vulnerable age	222	0.48	0.24–0.94	**0.03**	174	0.47	0.22–0.99	**<0.05**
Male	353	0.80	0.45–1.42	0.45	265	0.82	0.45–1.52	0.54
Smoking history	280	1.01	0.52–1.97	0.98	206	1.13	0.56–2.27	0.73
Adenoarcinoma	516	0.77	0.48–1.25	0.29	399	0.69	0.41–1.17	0.17
Poor differentiation	179	1.15	0.42–3.16	0.79	132	1.30	0.44–3.87	0.64
3 < tumor ≤ 4 cm	163	0.40	0.13–1.27	0.12	117	0.38	0.12–1.16	0.09
4 < tumor ≤ 5 cm	82	1.11	0.44–2.82	0.83	58	1.31	0.35–4.91	0.68
VPI	552	0.71	0.44–1.14	0.16	426	0.64	0.38–1.08	0.09
ELN < 10	86	0.87	0.28–2.72	0.81	61	0.79	0.21–2.99	0.73
LVI	50	0.40	0.04–3.77	0.42	37	0.00	0.00‐Inf	1.00
DFS								
Vulnerable age	204	0.63	0.40–0.99	**0.04**	163	0.68	0.43–1.08	0.11
Male	317	0.84	0.57–1.22	0.35	254	0.82	0.56–1.19	0.30
Smoking history	268	0.65	0.43–0.93	**0.04**	198	0.68	0.44–1.06	0.09
Adenoarcinoma	494	0.75	0.55–1.00	0.05	383	0.76	0.56–1.03	0.08
Poor differentiation	174	0.71	0.43–1.16	0.17	129	0.67	0.40–1.14	0.14
3 < tumor ≤ 4 cm	159	0.56	0.32–0.97	**0.04**	115	0.57	0.31–1.06	0.07
4 < tumor ≤ 5 cm	78	1.05	0.53–2.11	0.88	57	1.21	0.56–2.62	0.63
VPI	529	0.69	0.51–0.93	**0.01**	410	0.70	0.52–0.95	**0.02**
ELNs < 10	82	1.07	0.54–2.13	0.84	58	1.22	0.55–2.70	0.62
LVI	50	0.28	0.09–0.81	**0.02**	37	0.18	0.05–0.68	**0.01**

Bold numbers indicate statistical significance (*P* < 0.05). CI, confidence interval; DFS, disease‐free survival; ELNs, examined lymph nodes; HR, hazard ratio; LVI, lymphovascular invasion; N, number; OS, overall survival; PSM, propensity score matching: VPI, visceral pleural involvement.

To evaluate whether patients with different tumor sizes might benefit from ACT, we investigated the association between tumor size and five‐year OS or DFS rates among patients who either did or did not receive ACT. Consistent with previous studies, the association between small tumor size (0 < tumor ≤ 3 cm) and benefit from ACT was not confounded in this study. Moreover, patients in the 3 < tumor ≤ 5 cm group did not benefit from ACT, neither in the 3 < tumor ≤ 4 cm subgroup nor in the 4 < tumor ≤ 5 cm subgroup. The association between tumor size and benefit from ACT was not confounded regardless of PSM (Table 3). It appeared to be impossible to screen out the people who benefit from chemotherapy using tumor size alone; thus, further analysis is essential.

### Impact of ACT treatment on survival outcomes of stage IB–IIA NSCLC patients with VPI according to tumor size

**Figure 2:**
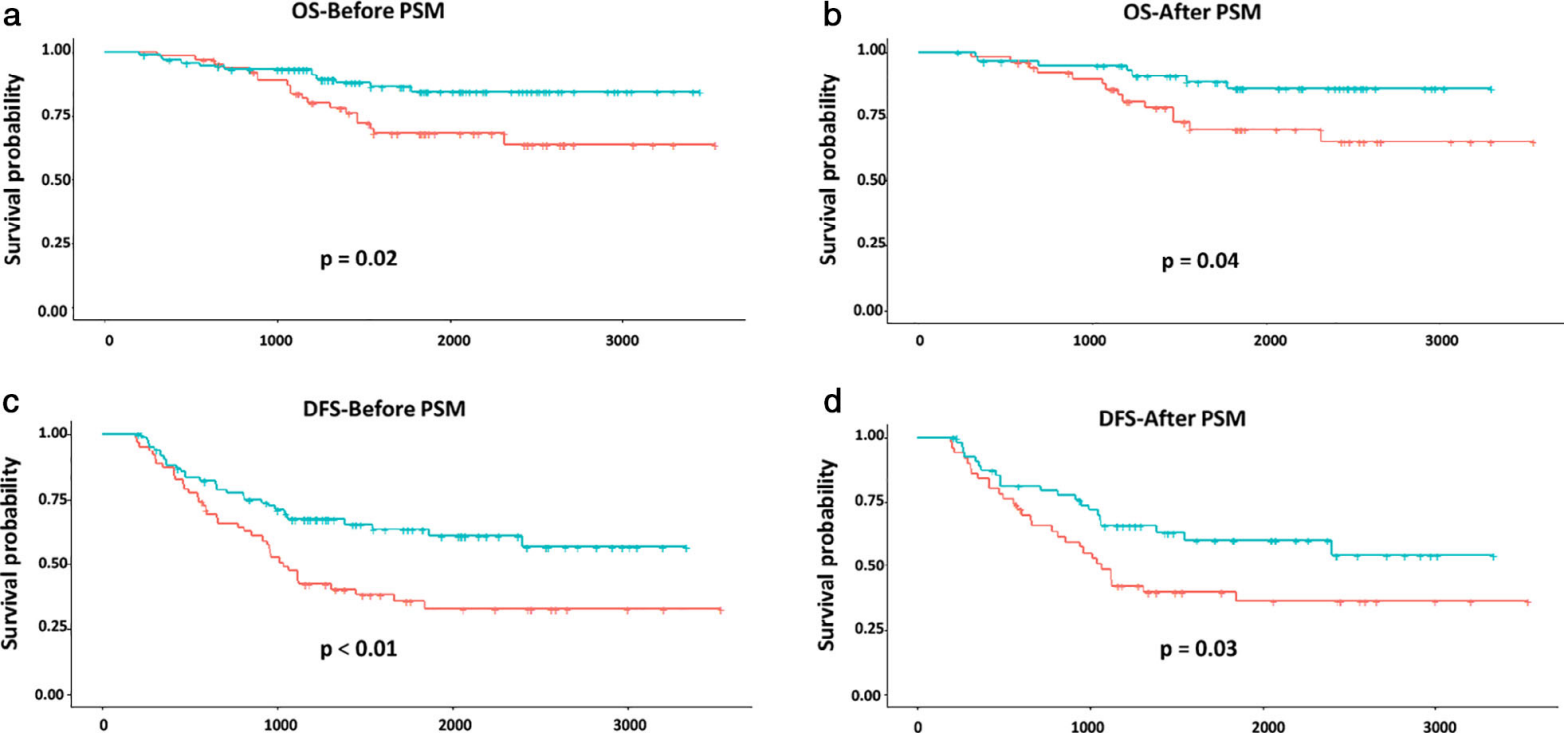
Kaplan‐Meier survival curves of OS and DFS for patients in the visceral pleural involvement (VPI) category in the 3 < tumor ≤ 5 cm subgroup following ACT treatment. (**a**,**b**) Kaplan‐Meier survival curves of OS for patients in the VPI category in the 3 < tumor ≤ 5 cm subgroup following ACT treatment before or after PSM 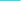, ACT; 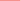, observation; 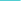, ACT; 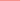, observation. (**c**,**d**) Kaplan‐Meier survival curves of DFS for patients in the VPI category in the 3 < tumor ≤5 cm subgroup following ACT treatment before or after PSM 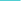, ACT; 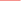, observation; 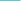, ACT; 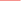, observation. ACT, adjuvant chemotherapy; DFS, disease‐free survival; OS, overall survival; PSM, propensity score matching.

To optimize triaging of patients for chemotherapy in stage IB–IIA NSCLC according to the eighth edition of the TNM classification, we investigated the effect of ACT treatment on survival outcome by different high risk in each tumor size subgroup. Interestingly, it seems that ACT conferred a survival benefit in patients with VPI in the 3 < tumor ≤ 5 cm subgroup. The five‐year OS rates in patients receiving ACT and those placed under observation were 84.58% and 68.10%, respectively (*P* = 0.02) (85.86% vs. 70.23%, *P* = 0.04, after PSM) (Fig 2a,b). Meanwhile, the five‐year DFS rates in these two subgroups were 63.33% and 35.62%, respectively (*P* < 0.01) (60.22% and 39.83%, *P* = 0.03, after PSM) (Fig 2c,d).

**Figure 3:**
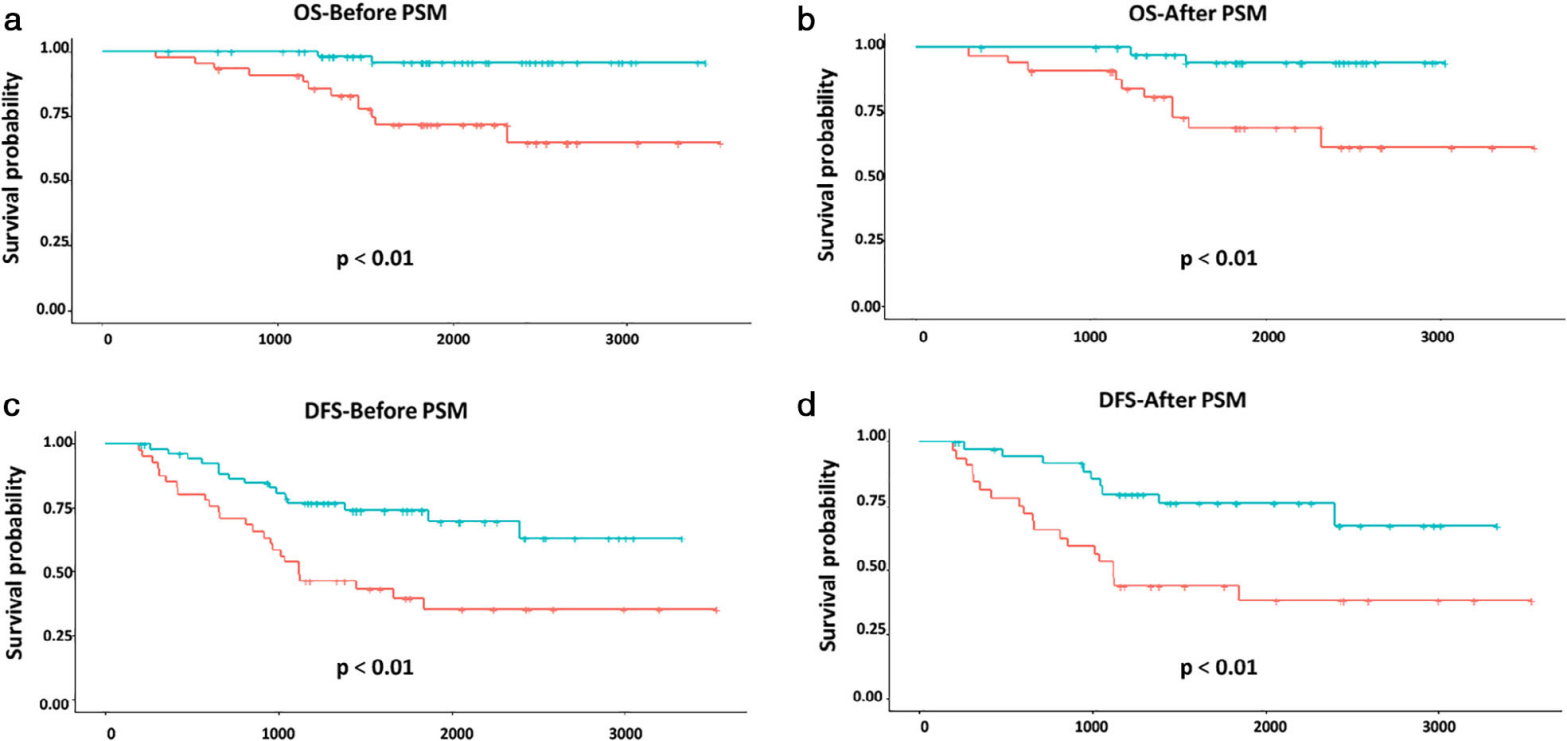
Kaplan‐Meier survival curves of OS and DFS for patients in the visceral pleural involvement (VPI) category in the 3 < tumor ≤ 4 cm subgroup following ACT treatment. (**a**,**b**) Kaplan‐Meier survival curves of OS for patients in the VPI category in the 3 < tumor ≤ 4 cm subgroup following ACT treatment before or after PSM 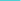, ACT; 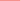, observation; 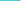, ACT; 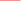, observation. (**c**,**d**). Kaplan‐Meier survival curves of DFS for patients in the VPI category in the 3 < tumor ≤4 cm subgroup following ACT treatment before or after PSM 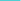, ACT; 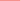, observation; 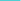, ACT; 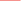, observation. ACT, adjuvant chemotherapy; DFS, disease‐free survival; OS, overall survival; PSM, propensity score matching.

Among such subjects, patients with VPI in the 3 < tumor ≤ 4 cm subgroup might benefit more from ACT treatment. The five‐year OS rates were 95.55% and 71.74% in the ACT and observation groups, respectively (*P* < 0.01) (93.98% and 68.93%, *P* < 0.01, after PSM) **(**Fig 3a,b **)**. In addition, the five‐year DFS rates were 74.01% and 39.65% in these two subgroups, respectively (*P* < 0.01) (75.90% and 43.75%, *P* < 0.01, after PSM) (Fig 3c,d). However, we did not to detect any benefit of ACT in NSCLC patients with VPI in the 4 < tumor ≤ 5 cm subgroup.

## Discussion

Although increasing evidence suggests that cisplatin‐based doublet ACT may improve OS for patients with surgical resection, whether stage IB–IIA NSCLC patients can benefit from ACT remains controversial.^16^, ^17^, ^18^, ^19^ Several studies regarding patients with completely resected NSCLC have demonstrated improved OS with ACT compared to observation. However, a large meta‐analysis included earlier results from JBR.10, CALGB9633, and IALT studies, demonstrating that there was no significant difference in OS between ACT and observation groups in the early stage patients after long‐term follow‐up.^6^, ^7^, ^8^, ^20^, ^21^ Only median DFS was notably better in the ACT group in the IALT study.^3^ In this study, we also did not detect an OS benefit of ACT in patients with stage IB–IIA NSCLC. However, further subgroup analysis showed that patients with VPI in the 3 < tumor ≤ 5 cm subgroup might benefit from ACT, particularly those in the 3 < tumor ≤ 4 cm subgroup.

In multiple studies, tumor size has been shown to predict outcome in NSCLC patients following surgical resection.^22^, ^23^, ^24^ As shown in subgroup analyses of the CALGB trial and several other trials, patients with tumor size greater than 4 cm benefit from ACT.^9^ In this study, the main inclusion criterion was pathological stage IB–IIA according to the eighth edition of the AJCC/UICC. Therefore, only those patients with tumor size <5 cm in diameter were included in the analysis. Some tumors that were previously stage IB were categorized as stage IIA (4 < tumor ≤ 5 cm). In concordance with most previous studies, patients with larger tumor size (4 < tumor ≤ 5 cm) had significantly worse OS and DFS than those with smaller tumor size. Unfortunately, ACT did not affect OS or DFS in such subgroups. The association between tumor size and ACT benefit was also not confounded in the other two subgroups (0 < tumor ≤3 cm; 3 < tumor ≤ 4 cm). Therefore, it appears unfeasible to identify those who benefit from chemotherapy based on tumor size alone.

It has been reported that VPI and tumor size have a synergistic effect on survival in node‐negative NSCLC. The prognostic value of VPI in stage IB NSCLC has been re‐evaluated using the prospective multicenter ACOSOG Z0030 trial data set.^25^ Stage IB patients with VPI and tumors >3 and ≤ 5 cm had significantly worse prognoses than those with T2a tumors. The meta‐analyses also disclosed the impact of VPI on node‐negative NSCLC patients. The data showed that VPI was a significant adverse prognostic factor in patients with tumor sizes >3, but ≤5 cm (OR 0.69, 95% CI: 0.56–0.86; *P* < 0.001).^26^ In addition, data from the California Cancer Registry suggested that VPI was an adverse prognostic factor along with tumor size with a greater significance in tumors >3 cm compared to smaller tumors. However, the role of ACT in stage IB–IIA NSCLC patients according to the eighth edition of the AJCC/UICC with VPI remains unclear. In this study, we demonstrated that patients with VPI might benefit from ACT because of the lower risk of cancer recurrence after ACT treatment. However, ACT had no effect on OS in these patients. Interestingly, patients with VPI in the 3 < tumor ≤ 5 cm subgroup might benefit from ACT. The five‐year OS and DFS rates were significantly higher than those in cases under observation. Moreover, in patients with VPI in the 3 < tumor ≤ 4 cm subgroup, ACT significantly reduced risk of death and recurrence (SI2). Potentially due to insufficient numbers, we failed to support a similar conclusion in the larger tumor size group (4 < tumor ≤ 5 cm). Thus, further multicenter studies are needed to confirm these findings.

Lymph node sampling or dissection plays an important role in precise nodal staging by identifying lymph node involvement and determining the extent of disease and the therapeutic effect on lymph node metastatic lesion clearance.^27^, ^28^, ^29^ The American College of Surgeons has endorsed removal of at least 10 lymph nodes as a quality metric in a well‐intended effort to improve care.^30^ Recently, Liang *et al*. reported that a greater number of ELNs is associated with more‐accurate node staging and better long‐term survival of patients with stage I–IIIA resected NSCLC.^31^ The study recommended 16 ELNs as the threshold for evaluating the quality of LN examination. Using the same threshold, we failed to confirm the association between the ELN number and NSCLC survival in this study, which may have been because of different populations in the two studies. The prior study used data from a Chinese multi‐institutional registry and the US SEER database on stage I to IIIA NSCLC. In the present study, only stage IB–IIA resected NSCLC cases were included. There was no statistical difference in OS or DFS between individual subgroups with 16 ELNs as the threshold (data not shown). Interestingly, having fewer ELNs (<10) was associated with a higher rate of recurrence. Moreover, we investigated whether patients with insufficient ELNs could benefit from ACT. Unfortunately, we were unable to conclude that the number of ELNs and ACT synergistically resulted in a better prognosis for patients with stage IB–IIA NSCLC; this may be because of the small number of cases with ELN < 10 included in this study. Further studies are required to clarify these potential associations. A more well‐defined standard for ELN number in early and advanced stage NSCLC may be needed.

We evaluated the influence of ACT on survival in stage IB–IIA NSCLC patients according to the eighth edition of the AJCC/UICC, which is the major strength of this study. Through further in‐depth analysis, we identified that only larger tumor size and advanced age were the potential independent prognostic factors. Having fewer ELNs was associated with a higher rate of recurrence. More importantly, patients with VPI in the 3 < tumor ≤ 5 cm subgroup might benefit from ACT, particularly so the patients in the 3 < tumor ≤ 4 cm subgroup.

This study also has certain limitations. First, it was a retrospective and single‐center study. Second, an insufficient number of cases and deaths were observed during the median follow‐up of 63.72 months in our cohort to fully investigate the interaction between ACT, and OS and DFS benefits in stage IB–IIA NSCLC patients following resection. A longer follow‐up period and multicenter approach may overcome this limitation.

In conclusion, ACT may improve DFS, but not OS, in patients with stage IB–IIA based on the eighth edition of the AJCC TNM classification. Although larger tumor size (4 < tumor ≤ 5 cm) was an independent prognostic factor, we did not conclude that patients in the 4 < tumor ≤ 5 cm subgroup could benefit from platinum‐based ACT. Fortunately, subgroup analysis confirmed that patients with VPI in the 3 < tumor ≤ 4 cm subgroup may benefit more from ACT. Further prospective randomized clinical trials are needed to further identify the role of ACT and better systemic strategies need to been developed in resected stage IB–IIA NSCLC.

## Disclosure

The authors declare that there are no conflicts of interest.
